# Platelet Rich Fibrin Matrix (PRFM) and Peripheral Blood Mesenchymal Stem Cells (PBMSCs) in the management of intraosseous defects - A randomized clinical trial

**DOI:** 10.1590/1678-7757-2023-0442

**Published:** 2024-08-05

**Authors:** R SREEPARVATHY, Sphoorthi Anup BELLUDI, Ashwin PRABHU

**Affiliations:** 1 K.L.E Society’s Institute of Dental Sciences Department of Periodontics Bengaluru India K.L.E Society’s Institute of Dental Sciences, Department of Periodontics, Bengaluru, India.

**Keywords:** Intrabony defect, Osseous regeneration, Peripheral blood mesenchymal stem cells (PBMSCs, Platelet-rich fibrin matrix (PRFM, Wound healing

## Abstract

A combination of peripheral blood mesenchymal stem cells (PBMSCs) and platelet rich fibrin matrix (PRFM) could be a probable periodontal regenerative material with the synergy of the added benefits of each material.

**Objective:**

This randomized controlled clinical trial aimed to evaluate the regenerative capacity of supercell (PRFM and PBMSCs) compared with that of PRFM alone in human periodontal mandibular intraosseous defects (IOD).

**Methodology:**

This study included 17 patients of both sexes (12 men, 5 women) aged 30-55 years (mean age = 37.7±4.4 years) who fulfilled the inclusion criteria (radiographic and clinical evaluation for bilateral IOD with probing pocket depth (PPD ≥ 6 mm). A split-mouth design was used in each patient. A total of 34 sites in the mandibular arch randomly received PRFM alone + open flap debridement (OFD) [Control sites] or supercell (PRFM+PBMSCs) + OFD [Test sites]. The clinical parameters plaque index (PI), gingival index (GI), PPD, clinical attachment level (CAL), and in the radiographic parameters; defect depth (DD) and defect fill percentage (DFP) were recorded at baseline, 3 and 6 months postoperatively. Early wound healing index (EHI) was used at 1 week to assess wound healing ability.

**Results:**

At 6 months, radiographic parameters revealed significant reduction in DD (P<0.001) and significant DFP values in the test group compared with the control group. The supercell showed significant improvement in PPD and CAL at the end of 6 months (P<0.001). EHI scores at 1 week showed no statistically significant difference between the test and control groups.

**Conclusion:**

Supercell can be considered a regenerative material in the treatment of periodontal IODs.

## Introduction

The continued quest to address the problem of periodontal regeneration and technological advancements have led to a plethora of platelet concentrates, which are a viable method to acquire growth factors (GFs).^1^ Extensive research has been carried out on Choukron’s Leukocyte-rich platelet rich fibrin (L-PRF) due to its autologous nature and advantages over platelet rich plasma (PRP).^2^

However, compared with PRF—a second-generation PC^3^, platelet rich fibrin matrix (PRFM), which belongs to the same generation and is also known as pure platelet rich fibrin (P-PRF) or leukocyte-poor platelet rich fibrin, is comparatively less researched. PRFM is an autologous biological substance obtained by double centrifugation of blood (without the addition of exogenous thrombin). As a result, the GF compliment is retained in the isolated viable intact platelets. Thus, when PRFM is applied to the wound site, there is a sustained, valuable release of GFs that influences osseous and soft tissue healing.^4^ Tissue repair is enhanced with the application of PRFM because it has the potential to preserve, isolate, and concentrate platelets within a dense fibrin matrix that acts as a scaffold.^5-8^

At the same time, advances in tissue engineering have highlighted the importance of mesenchymal stem cells (MSCs). The increasing focus on MSCs is due to their capacity for self-revival, proliferation, multipotent-lineage, and extended viability with extensive regenerative ability.^9^Among the sources of MSCs, peripheral blood (PB) MSCs have attracted attention because they are autologous, very convenient, and minimally invasively procured, unlike bone marrow-derived mesenchymal stem cells (BMMSCs), which are associated with complications such as chronic pain, hemorrhage, neurovascular injury, and even increased mortality.^10,11^ BMMSCs and PBMSCs share similar cellular, biological, and differentiation characteristics, which is helpful for soft and osseous tissue regeneration and repair.^12,13^ Although PBMSCs are less abundant, they can be detected and expanded to sufficient numbers for clinical efficacy and also engraft rapidly compared with BMMSCs.^14,15^

Therefore, incorporating PBMSCs into PC PRFM for periodontal regeneration would be more advantageous than using PRFM alone. To the best of our knowledge, no clinical studies have been conducted on human intrabony defects to investigate the regenerative capacity of this combination material, i.e., supercell (DiponEd Biointelligence LLP, Bengaluru, KA, India). Moreover, in this study, a modified PRFM kit (DiponEd Biointelligence LLP, Bengaluru, KA, India) with the incorporation of a patented gel was used in this study, eliminating the need for a second spin to procure the PRFM, which appears to be an added benefit.

Therefore, this study aimed to evaluate supercell as a regenerative substance compared with PRFM alone in periodontal IODs (three-wall defects) in the human mandibular region.

## Methodology

### Study design

This study was a single-center, double-blind, randomized, controlled clinical trial using a split-mouth design with a follow-up period of 6 months. Ethical approval for the study was obtained from the Institution’s Ethics Committee and Review Board (IEC and IRB) KIDS/IEC/NOV-2017/37, KLE Society’s Institute of Dental Sciences, Bangalore. The study was conducted in accordance with the Declaration of Helsinki, adopted by the 18^th^ World Medical Assembly in 1975 and revised in Edinburgh (2000). The study was conducted from December 2019 to November 2020 and registered with the Clinical Trials Registry - India (Register No. - CTRI/2019/11/022149).

### Study participants

The study participants were enrolled from the Department of Periodontics, KLE Society’s Institute of Dental Sciences, Bangalore, Karnataka. A total of 56 patients aged 30-55 years were included (Figure S1).

A complete history (dental and medical) was taken. Patients were then informed about the study and written informed consent was obtained. Patients were advised to take a routine hemogram (blood test) to assess random blood sugar (RBS), complete blood count (CBC), platelet count, differential count (DC), hemoglobin percentage (Hb%), clotting time (CT), and bleeding time (BT). Participants with underlying systemic diseases and those taking medications known to affect platelets or the outcome of periodontal therapy were excluded. Smokers, immunocompromised individuals, and pregnant or lactating women were excluded.

After 6 weeks of initial therapy, participants were evaluated and included based on the presence of bilateral mandibular PPD measuring ≥ 6mm with radiographic evidence of IOD and diagnosis of Stage III Grade B periodontitis. After evaluation of PPD and radiographs, only vital multi-rooted (molar) teeth with three-wall defects (assessed after surgical flap reflection) were included, resulting in 34 sites and 17 participants (mean age = 37.7±4.4 years) of both sexes (12 men, 5 women) (Table 1).

**Table 1:** Age and gender distribution among study subjects

Variable	Category	n	%
Age	30-35 yrs	6	35.30%
	36-40 yrs	7	41.20%
	41-45 yrs	4	23.50%
		Mean	SD
	Mean & SD	37.7	4.4
	Range	30-44	
Gender	Male	12	70.60%
	Female	5	29.40%

R S, Belludi SA, Prabhu A

### Initial therapy

Patient enrollment was conducted by a single therapist. After a preliminary examination and discussion of the treatment plan, all selected patients received detailed instructions on plaque control measures and underwent initial periodontal therapy consisting of scaling and root planing (SRP). Occlusal analysis and adjustments were performed as indicated. Oral hygiene and tissue response were re-evaluated 4-6 weeks after SRP, and clinical and radiographic values were recorded.

### Randomization, allocation, and interventions

Sites were randomly assigned to the test (supercell - PRFM+PBMSCs - Material A) or control (PRFM alone - Material B) groups using a computer-generated tabulation method (Figure S1). Allocation was performed using sealed opaque envelopes. The allocation sequence was concealed until the intervention. Patients who met all criteria were included and proceeded to the surgical phase of therapy. Once the surgeon/operator (SR) informed that the reflected site had a three-wall defect, the periodontist (AP) selected an envelope to know and procure the material (supercell - PRFM+PBMSCs - Material A or PRFM alone - Material B) designated in the envelope. At the subsequent surgical visit, the periodontist (AP) procured the remaining material to be tested on the other side of the arch (split mouth). The right-side defects were operated on first, regardless of whether the sites received Material A or Material B after randomization. The subsequent site was operated on two weeks later. All therapeutic procedures were performed by a periodontal surgeon/operator (SR), and another examiner (SAB) performed all clinical measurements. The periodontal surgeon/operator, examiner, and participants were blinded to the study groups and materials used.

### Primary outcome measures

Defect depth (DD) and defect (bone) fill percentage (DFP) were recorded at baseline and 3 and 6 months postoperatively.

### Secondary outcome measures

Probing Pocket Depth (PPD) and Clinical Attachment Level (CAL) were measured at baseline and 3 and 6 months postoperatively.

Plaque Index (PI) and Gingival Index (GI) were measured at baseline and 3 and 6 months postoperatively.

Wound healing was measured using the wound healing index (WHI) 1 week postoperatively.

Preoperative clinical measurements:

PI (Tureskey modification of Quigley Hein Index) and GI (Loe and Silness) were measured at 6 aspects per tooth, i.e., mesiobuccal, midbuccal, distobuccal, mesiolingual, midlingual, and distolingual.^16^

To measure the probing pocket depth, a custom cold cure acrylic resin stent was fabricated for each patient on their study model over the selected test region to provide a reproducible fixed reference point for reducing measurement error.^17^ A groove was cut in this stent to allow a manual periodontal probe (University of North Carolina -15 probe) to be inserted at a standardized pocket entry point preoperatively and postoperatively.

**Figure 1:**
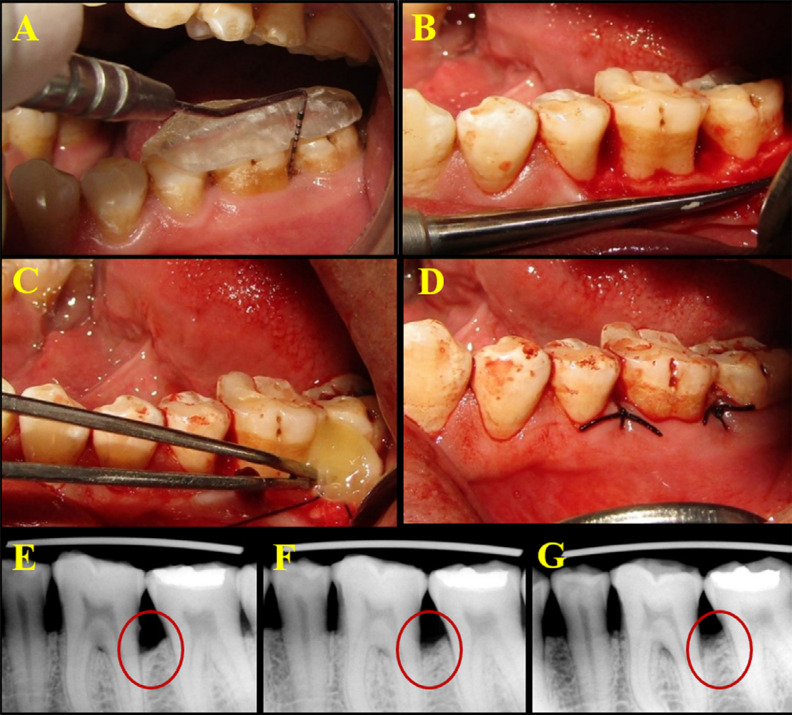
Clinical procedure of PRFM placement and radiographic images of follow-up at baseline, 3 months, and 6 months. 1A – Preoperative clinical PPD; 1B – Full thickness flap reflection and debridement of the defect site; 1C – Placement of PRFM into the defect site; 1D – Site sutured; 1E – Radiographic images at baseline, 3 months, and 6 months

Pocket depth was measured from the crest of the gingival margin to the base of the pocket and rounded to the nearest millimeter for recording on the customized and personalized case proforma (Figure 1A, 2A). The customized acrylic stents on the prepared study casts were stored in water to minimize distortion during the study period.^18^

**Figure 2:**
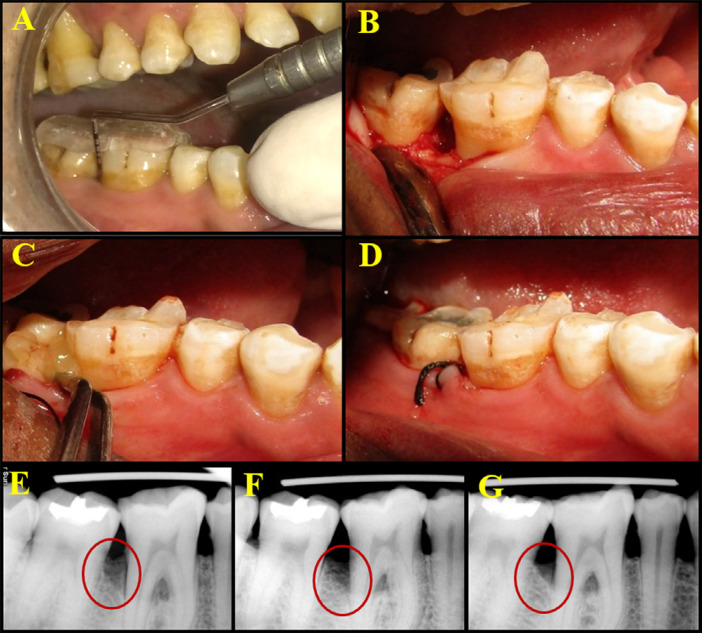
Clinical procedure of supercell placement and radiographic images of follow-up at baseline, 3 months, and 6 months. 2A – Preoperative clinical PPD; 2B – Full thickness flap reflection and debridement of the defect site; 2C – Placement of supercell into the defect site; 2D – Site sutured; 2E – Radiographic images at baseline, 3 months, and 6 month

After positioning the occlusal acrylic stent, the periodontal probe was gently inserted through the cut groove. The level of attachment was measured as the distance between the base of the pocket and the fixed reference point (CEJ).^19^

### Radiographic measurements:20

An intraoral direct digital periapical radiovisiograph (Suni Medical Imaging, Apteryx Inc., Acron, Ohio, USA) of each defect site was taken using a long cone paralleling technique. Exposures were made at 70 KVp, 7 ma for 0.2 seconds. The focus-to-film distance was 20 cm, and a film holder^||^ (Troll byte plus, Troll Dental, Trollhattan, Sweden) was used.

To minimize errors, the radiographic images were taken by the same examiner (SAB) at baseline and 3 and 6 months postoperatively. A 1x10mm orthodontic wire incorporated into the customized occlusal stent allowed for estimation of magnification.

Linear measurements were made on the digitized images (Figure S6) using analysis software (Image J). For each defect, the following two distances were assessed by RVG:

- Cementoenamel junction (CEJ) to Alveolar crest (AC)
- Cementoenamel junction (CEJ) to Base of defect (BOD)

Preoperative measurements at baseline:

A – CEJ to the base of the defect (BOD)

B – CEJ to the alveolar crest (AC)

C – Defect depth at baseline (A - B)

Postoperative measurements at 3 months:

A 1 – CEJ to BOD

B 1 – CEJ to AC

C 1 – Defect depth at 3 months (A1 ± B1)

D – Defect fill in mm (C ± C1)

Postoperative measurements at 6 months

A2 – CEJ to BOD

B2 – CEJ to AC

C2 – Defect depth at 6 months (A2 ± B2)

D1 – Defect fill in mm (C ± C2)

Percentage of defect fill:


$$\frac{\text{ Baseline defect depth }-3/6\text{-month defect depth }\times 100}{\text{ Baseline defect depth }}$$

### Surgical procedures

The surgical site was anesthetized with 2% Xylocaine HCl with adrenaline (1:80,000). After achieving adequate anesthesia, crevicular incisions were made on the facial and lingual/palatal sides up to the tip of the interdental papilla using a B.P. handle with a no.12 blade, and an interdental incision was made with a no.15 blade. A periosteal elevator was used to reflect a full-thickness mucoperiosteal flap, taking care to preserve the interdental papillary tissue as much as possible. After reflection of the flap and exposure of the osseous defect, a thorough surgical degranulation of the infected tissue from the osseous defects was performed using Gracey curettes and 4R-4L Columbia, Universal Curettes (Hu-Friedy, USA). Thorough root planing was performed and the surgical site was then thoroughly irrigated with normal saline (Figure 1B, 2B).

The following sequential steps were followed for the fabrication of PRFM^21^ (Figure S2):

1. 9 ml of patient blood was drawn into the Merisis PRFM tube.
2. The tube was placed in a centrifuge (REMI-8C, REMI, India) and spun at 3400 rpm for 5 minutes to separate the blood into supernatant plasma and platelet suspension.
3. After centrifugation, the final product obtained was PRFM.

The following sequential steps were followed for the procurement of PBMSCs embedded PRFM:

1. First, stem cells were prepared from 9 ml of patient blood drawn into the Merisis supercell tube.
2. The tube was then placed in a centrifuge with a swing bucket rotor and spun at 3400 rpm for 6 minutes to separate the blood into supernatant plasma and stem cell suspension.
3. After centrifugation, RBCs were located below the cell separator gel, and stem cells, 0.5-1 ml above the gel, was kept aside for further use. The platelet-poor plasma (PPP) above the stem cells was aspirated and discarded.
4. 8 ml of patient blood was drawn again and mixed with 1 ml of the previously prepared stem cells.
5. The tube was then placed in a centrifuge and spun at 3400 rpm for 5 minutes to separate the blood into supernatant plasma and platelet suspension.
6. After centrifugation, due to fibrin polymerization, supercell, which is the combination of stem cells and PRFM, was formed and ready for use.

PRFM was placed in the control site (Figure 1C) and supercell was placed in the test site (Figure 2C), after which the pre-sutured mucoperiosteal flaps were approximated and periodontal dressing was applied. (Figures 1D and 2D). Postoperatively, patients were advised to apply cold compresses to reduce edema. The postoperative medications prescribed to patients included antibiotics and analgesics. The antibiotics prescribed were amoxicillin 500mg three times a day for 5 days. Patients allergic to penicillin were prescribed erythromycin 500 mg. Patients were recommended to take analgesics (ibuprofen 400 mg three times a day) and antiseptic oral rinse (0.2% chlorhexidine gluconate mouthwash two times a day) for a week.^22^

### Immediate postoperative care

One week after surgery, the periodontal dressing and sutures were removed and the area was thoroughly irrigated with saline. Patients were asked about discomfort, pain, and sensitivity. A recall appointment was scheduled at 1 week and postoperative healing was assessed using EHI differentiating between 5 different degrees.

### Statistical analysis

#### Sample Size Estimation

The sample size for the study was estimated using a sample size calculation software (G Power software version 3.1.9.2). Considering the effect size (f) as 25%, the power of the study as 80%, and the margin of α error as 5%, the total sample size was calculated to be 34. Thus, each of the two groups consisted of 17 intrabony defect sites.

The values obtained from clinical and radiographic evaluation were tabulated and analyzed using a statistical software (SPSS version 16.0). Student ‘t’ test was used for the intergroup comparison. ANOVA with Greenhouse-Geisser correction and post-hoc tests were used for the intragroup comparison. The intra-class correlation coefficient (ICC), used to assess intra-examiner consistency, revealed a high level of agreement (ICC=0.9).

## Results

A total of 34 intrabony sites in 17 patients were evaluated. No patients dropped out of the study (Figure S1). For the primary outcome measure, the defect depth in 1/10th of a mm was analyzed for the specific sites and compared at the specific follow-up time points between the two study groups.

Friedman’s test was used for intragroup comparisons at different time points for all clinical parameters. Pairwise comparisons between different time points in each group were performed using the Wilcoxon signed rank post-hoc analysis.

Intragroup comparisons of different time points:

The intragroup comparison of the defect depth within each of the two groups showed statistical significance in the test group (P<0.001). In both the control and test groups, the gradual decrease in the mean defect depth from baseline to 3 months, baseline to 6 months, and 3 months to 6 months was highly statistically significant (P<0.001) (Tables 2 and 3, Figure S4). The mean defect fill percentage was calculated at both 3-month and 6-month intervals. In the control group, the mean defect fill percentage was calculated to be 19.15±13.98% at 3 months and 45.20±15.95% at 6 months. In the test group, it was calculated to be 41.66±13.90% at 3 months and 63.52±14.86% at 6 months. (Table 2, Figure S4).

**Table 2:** Comparison of mean clinical parameters between control and test sites at baseline, 3 months, and 6 months using Wilcoxon signed rank test

Variable	Site	N	Mean			SD			Mean Diff			P-value		
			BL	3M	6M	BL	3M	6M	BL	3M	6M	BL	3M	6M
PI	Control	17	2.32	1.27	1.81	0.45	0.79	0.69	-0.04	0.01	1.21	0.82	0.95	<0.001*
	Test	17	2.37	1.25	0.6	0.49	0.56	0.67						
GI	Control	17	2.37	1.25	1.62	0.49	0.56	0.8	-0.15	-0.01	0.96	0.31	0,95	0.002*
	Test	17	2.52	1.27	0.66	0.42	0.79	0.66						
PPD	Control	17	8.12	5.88	4.94	1.22	1.11	0.66	-0.41	0.23	0.59	0.19	0.04*	0.001*
	Test	17	8.53	5.65	4.35	1.5	1.11	0.61						
CAL	Control	17	7.82	6.35	5.12	2.6	1.84	0.93	-0.41	1.06	0.88	0.31	0.003*	0.001*
	Test	17	8.24	5.29	4.24	3.27	1.57	0.83						
Defect Depth	Control	17	67.63	56.57	39.5	25.64	24.54	20.43	-10.9	10.64	11.33	0.08	0.02*	0.009*
	Test	17	78.53	45.93	28.17	38.76	23.53	15.76						
Defect Filling	Control	17	-	19.15	45.2	-	13.98	15.95	-	-22.51	-18.33	-	0.001*	<0.001*
	Test	17	-	41.66	63.52	-	13.9	14.86						

*- Statistically Significant; PI,GI-scores; PPD, CAL, DD – mm; DF - %

**Table 3:** Comparison of mean defect depth between different time intervals in the control and test sites using Friedman’s test followed by Wilcoxon signed rank post-hoc analysis

Site	Time	N	Mean	SD	P-Value^**a**^	Sig. Diff	P-Value^**b**^
Control	Baseline	17	67.63	25.64	0.001*	BL vs 3M	<0.001*
	3 Months	17	56.57	24.54		BL vs 6M	<0.001*
	6 Months	17	39,5	20.43		3M vs 6M	<0.001*
Test	Baseline	17	78.53	38.76	<0.001*	BL vs 3M	<0.001*
	3 Months	17	45.93	23.53		BL vs 6M	<0.001*
	6 Months	17	28.17	15.76		3M vs 6M	<0.001*

*- Statistically Significant

A statistically significant reduction in PPD and gain in CAL were observed from baseline to 3 and 6 months in both the control and test groups (P<0.001). The intragroup comparison of site-specific PI and GI scores in both the control and test groups at different follow-up time points revealed a statistically significant difference (p<0.001). Pairwise comparison between the groups at different time points showed that PI and GI scores were highest at baseline, followed by 3 months, and lowest at 6 months. (Table 2, Figure S3)

Comparison of outcome measures between the two groups:

The intergroup comparison of mean defect depth was performed using the Wilcoxon signed rank test at each of the time points. While there was no statistically significant difference between the two groups at baseline, there was a statistically significant difference at 3 and 6 months (P<0.001), with the test group showing a significantly lower mean defect depth than the control group (Table 2).

The intergroup comparison of the mean defect fill percentage between the two groups at 3 and 6 months showed a statistically significant difference (P<0.001), with the test group having a significantly higher mean defect fill percentage than the control group (Table 2). The reduction observed in PPD from baseline to the end of 3 and 6 months was statistically significant (P<0.001) in the test group (8.53±1.50mm, 5.65±1.11mm, and 4.35±0.61mm at baseline, 3 months, and 6 months respectively) compared with the control group (8.12±1.22mm, 5.88±1.11mm, and 4.94±0.66mm at baseline, 3 months, and 6 months respectively) (Table 2, Figure S3). The intergroup comparison of mean CAL showed that the groups were comparable at baseline, but there was a statistically significant difference at 3 and 6 months (P<0.001), with the test group having a much lower mean CAL (8.24±3.27, 5.29±1.57, and 4.24±0.83 at baseline, 3 months, and 6 months respectively) than the control group (7.82±2.60, 6.35±1.84, and 5.12±0.93 at baseline, 3 months, and 6 months, respectively) (Table 2, Figure S3).

The mean PI and GI scores at baseline and 3 months were comparable between the groups, but at 6 months there was a statistically significant difference, with the test group having much lower mean scores than the control group (P<0.001) (Table 2, Figure S3).

Early wound healing index scores at 1 week showed no statistically significant difference between the test and control groups (Table 4, Figure S5).

**Table 4:** Comparison of EHI scores between control and test sites at 1 week using McNemar’s test

EHI	Control Site		Test Site		P-Value
	n	%	n	%	
Score 1	14	82.40%	15	88.20%	0.9
Score 2	3	17.60%	2	11.80%	

R S, Belludi SA, Prabhu A

## Discussion

In recent years, treatment concepts in periodontology have aimed to develop, apply, and analyze a highly predictable material in clinical practice for periodontal tissue regeneration and functional attachment close to that of the natural tooth.

PCs are autologous biological products derived from the patient’s whole blood and consist mainly of supraphysiologic consolidation of platelets and PDGF. PRFM is one of the several known PCs with proven regenerative potential. Studies have shown that PRFM can be a clinically significant periodontal regenerative material in the treatment of vertical intraosseous defects.^23^The crosslinking of fibrin that occurs within the procured PRFM stabilizes the clot, prevents retraction, and creates a consistency that resists displacement, maintains space, and thereby inhibits soft tissue invasion, thus enhancing its properties.^4-7^

Stem cells are characterized by their potential for continuous self-renewal and their ability to differentiate into a specialized adult cell type. In contrast to the routinely used bone marrow, PB can be easily obtained as a source of MSCs. Therefore, PBMSCs serve as a promising source for bone regeneration for clinical use.^24^ In our study, we procured PRFM and PBMSCs using the Merisis kit (DiponEd Biointelligence LLP, Bengaluru, KA, India). The Merisis kit (DiponEd Biointelligence LLP, Bengaluru, KA, India) offered the advantage of procuring PRFM with a single spin rather than double centrifugation. This provided an advantage over conventional PRFM preparation, which requires considerable time and effort.^4^ Similarly, PBMSCs were also amassed and expanded to adequate numbers, maintaining their osteogenic potential within a clinically favorable time frame (Belludi, et al.^25^ [2021]).

Therefore, the intention to combine the beneficial properties of PRFM and PBMSCs (Supercell) for regeneration would be unique and may be more beneficial than using PRFM alone. The periodontal tissue response to supercell was evaluated by recording EHI and various clinical and radiographic parameters, such as PI, GI, PPD, CAL, and osseous defect fill percentage.

The randomized split-mouth design used in the study eliminates the influence of patient-specific characteristics and facilitates the interpretation of trials by minimizing the effects of interpatient variability.^26^ The sample size used in this study is relatively small, but is within the range adopted by the majority of clinical periodontal regenerative studies in humans.^27^

The sites selected in the study included mandibular periodontal IODs due to the increased prevalence of such defects in the mandibular arch, as reported by Singh and Kumari^28^ (2017) in a sample of the Indian population.

In our study, supercell application was well accepted by all the patients. No signs of discomfort, infection, and/or other unfavorable reactions were reported by patients at any stage of the study. A similar type of well-tolerated patient response was reported with the application of PRFM in extraction sockets by Simon, et al.^29^(2011).

The inconsistency associated with clinical periodontal probing in this study was reduced by the use of custom acrylic stents with guiding grooves for reproducibility of probing directions and sites as described by Rams and Slots^18^ (1993). It was concluded that easily identifiable reference points provided by custom acrylic splints are very convenient for systematizing the probing angulation and probe insertion site.^30^

The intragroup comparison within the two groups revealed a statistically significant difference in the mean PI and GI scores at the different time points (P<0.001), and the significance was greater in the test group. In the control group, the decrease in mean PI scores from baseline to 3 and 6 months was highly statistically significant. In the control group, there was a significant decrease in mean GI scores from baseline to 3 and 6 months. This decrease can be attributed to the reduction in PI scores and the consequent reduction in gingival inflammation. The intergroup comparison of mean PI and GI scores, performed using the Wilcoxon signed rank test at each of the time points, revealed that there was a statistically significant difference between the two groups at 6 months (P<0.001) (Table 2, Figure S3).

This suggests that all patients in this split-mouth study maintained adequate oral hygiene throughout the study. In addition, previous studies have revealed that various PCs have antibacterial activity, which may also have contributed to the reduction in mean PI scores.^31^ Studies have also shown that MSCs have antibacterial activity.^32^ There was also a significant difference between the two groups at 6 months, suggesting that the addition of PBMSCs enhanced the effects of PRFM on plaque. This invariably affected the reduction in GI scores. A combined effect subsequently resulted in some degree of post-healing tissue shrinkage and a reduction in PPD.^33^

The intragroup comparisons revealed a significant difference in the mean PPD at different time points. The intergroup comparison of PPD showed a statistical reduction in PPD from baseline to the end of 3 and 6 months in the test group compared to the control group (P<0.001). (Table 2, Figure S3). The primary reasons for the reduction in PPD after the treatment were the reduction in inflammation and shrinkage of the pocket wall. This reduction also occurred due to the combined effect of the gain in CAL and post-treatment gingival recession.^34^The results of the mean PPD, considering its gingival component (with reference to the mean GI scores), suggest that both treatment approaches were effective in reducing the mean PPD from baseline to 6 months, while the PBMSCs had an additional benefit on the effect of PRFM. Similar to the results of this study, Patel, et al.^35^ (2017) demonstrated significant soft tissue healing and reduction in PPD in the group treated with PRF compared with that treated with OFD.

The intragroup comparisons showed a significant difference in the mean CAL at various time points, with the significance being greater in the test group (P<0.001). The intergroup comparison at 3 and 6 months showed a significant difference, with the test group having a much lower mean CAL than the control group (Table 2, Figure S3). The mean CAL results suggest that while both treatment modalities were effective in reducing mean CAL from baseline to 6 months, the addition of PBMSCs to PRFM provided additional regenerative potential.

This study showed no statistically significant difference in index scores between the test and control groups, with the majority of sites in both groups having a score of 1 at the end of one week (Table 4, Figure S5). This suggests that PRFM contributed to superior postoperative wound healing in both treatment modalities, confirming that PRFM alone contributed to soft tissue healing and that the addition of PBMSCs did not provide statistically significant added benefits. Similar to these results, previous studies have demonstrated acceleration of tissue regeneration by stimulating normal physiology.^8,29,36^ As indicated by various studies, the viable platelets in PRFM, which contain intrinsic growth factors such as platelet-derived growth factor, and the fibrin matrix, which acts as a scaffold for migrating endothelial cells, osteoblasts, and other cells required for tissue repair, may account for the results of our study.^8,37,38^ A clinical trial using PRF showed similar outcomes, with WHI results being more significant in the PRF group than in the OFD group.^35^

As we observed commensurate significant results correlating PI, GI, PPD, and CAL in the test group, it can be concluded that the addition of PBMSCs to PRFM enhanced and contributed not only to periodontal regeneration but also to the resolution of inflammation. In a systematic review, Fabbro, et al.^32^ (2016) concluded that autologous platelet concentrates have antimicrobial properties and are beneficial in surgical sites postoperatively.^31^Similarly, a study by Sung, et al.^33^ (2016) demonstrated that MSCs play a seminal role via anti-inflammatory and antibacterial effects.^32^

To estimate magnification, an occlusal splint with a known diameter (1mm) and length (10mm) of orthodontic wire was customized as described by Talaiepour, et al.^39^ (2005). RVGs were taken by the same clinician throughout the study to minimize errors.

Osteoneogenesis is often considered a primary outcome variable in controlled clinical trials of regenerative therapy. Radiographic assessment of alveolar bone changes is a non-invasive, painless alternative to surgical re-entry after regenerative procedures.

The intragroup comparisons of DD revealed a gradual decrease from baseline to 6 months, with differences of high statistical significance between the different time points, a significance that was greater in the test group (P<0.001) (Table 3). The intergroup comparison revealed a significant difference between the two groups at 3 and 6 months post-surgery (Table 2, Figure S4). Thus, it can be inferred that while both treatment modalities effectively reduced the intraosseous defect depth, the addition of PBMSCs to PRFM seemed to enhance the effect of the latter at 6 months follow-up period.

The intragroup comparisons of mean DFP showed a high statistical significance between 3 and 6 months (P<0.001). Intergroup differences in DFP were also statistically significant at 3 and 6 months, with the test group showing a much greater mean DFP than the control group (P<0.001) (Table 2, Figure S4). This along with the results of DD suggests that, while both materials contribute to gradual and progressive defect fill postoperatively up to 6 months, the incorporation of PBMSCs into PRFM appears to enhance and magnify the effect of the latter.

Consistent with these findings, a canine study conducted a histological evaluation of extraction socket healing and found that PRFM produced rapid healing with greater osseous fill than non-viable materials (DFDBA).^40^

Although animal studies provide extensive evidence that MSCs can be used effectively and safely for periodontal regeneration, very few human trials have been conducted and none have used PBMSCs in intraosseous defects. However, some studies have used MSCs of dental origin (periodontal ligament, pulpal, and other sources). An RCT by Chen, et al.^41^ (2016) concluded that periodontal ligament stem cells showed safety, efficacy, and promising results in the treatment of intraosseous defects. Consistent with this, our study not only demonstrates the same, but also shows that the addition of PBMSCs to PRFM has indeed demonstrated significant and safe results in terms of osseous defect fill and percentage, showing an additional primacy in osseous regeneration, with PRFM acting as a scaffold for the incorporation of PBMSCs. Concurrent with these results, a study on calvarial bony defects demonstrated that peripheral blood MSCs can be induced into multilineage cells, including osteoblasts. The study provided solid evidence that transplanted PBMSCs survived in the bony defect area and directly contributed to new bone formation.^42^A systematic review and meta-analysis by Miron, et al.^43^ (2021) concluded that the use of PCs (PRF) improved PD CAL and radiographic bone fill compared with OFD, and similar results were found for OFD+Bone grafts, OFD+emdogain, and OFD+Barrier membranes. The benefit of adding PBMSCs to the already substantiated PC was demonstrated in a randomized controlled clinical trial conducted by Singhal, et al.^21^ (2022), who reported that PRFM and PBMSCs showed improved bone-to-implant contact.

In addition, PRFM and PBMSCs are viable and biocompatible autologous biological materials with the added advantage that this procedure does not require the manipulation and placement of a membrane or the incorporation of other non-vital graft materials, support by itself as a scaffold, acting as a membrane. The benefits of not using a membrane include a faster and less complicated surgical procedure and the elimination of problems associated with infection, membrane movement, and membrane exposure. These aspects highlight the advantages of combining these two materials. However, this study has some limitations, including the inability to assess the nature of the regenerated periodontal tissues by histological examination, due to ethical constraints, and the lack of consideration of the extent of possible gingival recession as a consequence of the surgical approach used.

## Conclusion

The incorporation of PBMSCs into PRFM is an advantageous approach, and the synergistic action of the two materials demonstrates results towards periodontal regeneration, improvement of clinical parameters, and resolution of inflammation. However, multicentric clinical trials with prospective, double-blind, randomized controlled trials with larger sample sizes and long-term follow-up should be conducted in the near future to further assess the potential of supercell (PRFM and PBMSCs) in the surgical management of periodontal intraosseous defects.
